# Assessing the impact of COVID-19 on outpatient psychiatric population well-being and symptomology utilizing COVID-19 Events Checklist (CEC) and Measurement Based Care

**DOI:** 10.1186/s41687-024-00802-z

**Published:** 2024-11-21

**Authors:** Sydney B. Jones, Hayoung Ko, Alyssa J. Gatto, Anita S. Kablinger, Hunter D. Sharp, Lee D. Cooper, Martha M. Tenzer, Virginia C. O’Brien, Robert S. McNamara

**Affiliations:** 1 Department of Psychology, Virginia Tech, Blacksburg, VA 24060 USA; 2 Department of Psychiatry, University of Colorado Anschutz School of Medicine, Aurora, CO 80045 USA; 3 Children’s Hospital Colorado, Aurora, CO 80045 USA; 4 Department of Psychiatry and Behavioral Medicine, Virginia Tech Carilion School of Medicine, Roanoke, VA 24016 USA; 5 Health Analytics Research Team (HART), Carilion Clinic, Roanoke, VA 24011 USA

**Keywords:** COVID-19, Pandemic, Patient Reported Outcome Measures (PROMs), Coping, Mental health, Psychiatry, Measurement Based Care, Crisis Response, Routine Outcome Monitoring

## Abstract

**Background:**

This study examines the impact of SARS-CoV-2 (i.e., coronavirus, COVID, COVID-19) using data from a measurement-based care (MBC) system utilized in an outpatient psychiatric clinic providing telemedicine care. A novel Patient Rated Outcome Measure (PROM), the COVID-19 Events Checklist (CEC) was administered in a hospital system based ambulatory clinic beginning April 2020 to track COVID-19-19’s impact on patients’ mental, emotional, and health-related behaviors during the pandemic. The study (1) provides descriptive CEC data, and (2) compares CEC results with PROMs evaluating anxiety (Generalized Anxiety Disorder-7; GAD-7), depression (Patient Health Questionnaire; PHQ-9), and psychological distress (Brief Adjustment Scale-6; BASE-6).

**Methods:**

This retrospective observational study included patient intake data collected from April 2020 to March 2021. Patient (*N* = 842) reports on the CEC’s five domain questions were aggregated to calculate average reports of COVID-19 related impacts at intake over the initial 12 months of the pandemic. Trends in COVID-19 related impacts were examined, and non-aggregated scores on the PHQ-9, GAD-7, and BASE-6 were compared to primary dichotomous (yes/no) CEC survey questions via Wilcoxon rand sum testing.

**Results:**

Results capture the relationship between COVID-19 exposure, COVID-19- related sequelae and behaviors, and psychological symptom severity. Specifically, Wilcoxon rank-sum tests indicate that social determinants of health (SDOH), negative mental health impacts, and positive coping skill use were significantly associated with psychological symptomatology including overall psychological functioning via the BASE-6, anxiety via the GAD-7, and depressive symptoms via the PHQ-9. Results regarding SDOH were as follows: BASE-6 (w = 44,005, *p* < 0.001), GAD-7 (w = 44,116, *p* < 0.001), and PHQ-9 (w = 43,299, *p* < 0.001). Regarding negative mental health outcomes, the results were: BASE-6 (w = 38,374, *p* < 0.001), GAD-7 (w = 39,511, *p* < 0.001), and PHQ-9 (w = 40,154, *p* < 0.001). As the initial year of the pandemic elapsed, incoming patients demonstrated increased rates of suspected or confirmed exposure to COVID-19, (+2.29%, t = 3.19, *p* = 0.01), reported fewer negative impacts of COVID-19 on SDOH (−3.53%, t= −2.45, *p* = 0.034), and less engagement in positive coping strategies (−1.47%, t = −3.14, *p* = 0.010).

**Conclusions:**

Psychosocial factors related to COVID-19 are discussed, as well as opportunities for further research on the relationship between psychological symptomatology and the impact of COVID-19 on health-related behaviors.

**Supplementary Information:**

The online version contains supplementary material available at 10.1186/s41687-024-00802-z.

## Background

Measurement Based Care (MBC) [21] is known to improve the quality of mental health treatment via the use of Patient Reported Outcome Measures (PROMs). Since the start of the COVID-19 (COVID) pandemic, there has been increased interest in capturing the impact of pandemic-related stressors. The COVID-19 Events Checklist (CEC) [16] is a measure designed to do so. While a plethora of studies examine the influence of the ongoing pandemic in the years since 2020, few have examined use of tools specifically created to measure the impact of pandemic-related factors on incoming psychiatric patients [9, 11, 20, 28]. Studies have found that non-psychiatric populations have reported higher rates of psychosocial symptomatology including loneliness, severe anxiety, harmful drug use, and alcohol dependency as the COVID-19 pandemic has evolved [14]. In those with suspected or confirmed exposure to COVID-19, negative mental health outcomes were demonstrated across varied racial, socioeconomic, and clinical diagnostic groups [6, 29]. While the negative mental health outcomes associated with COVID-19 have been shown to have a disproportionate impact on individuals with preexisting mental health diagnoses, people of color, and those in low to middle income countries, the use of telehealth and telepsychiatry has been shown to decrease the impacts of pandemic stressors [13, 26, 27]. Many studies to date have examined COVID’s impact through the lens of PROMs also employed in the current study, including the General Anxiety Disorder-7 (GAD-7) [30], Patient Health Questionnaire (PHQ-9) [19], and Brief Adjustment Scale-6 (BASE-6) [8]. Few studies have utilized the CEC which was developed and initiated as a PROM in April 2020 following the commencement of the pandemic. In a virtual setting necessitated by the COVID-19 pandemic, PROMs have been useful in helping maintain therapeutic alliance, improving mental health outcomes, and tracking the impact of the pandemic on patients’ mental well-being as a secondary function of MBC.

The present study utilized the CEC along with the aforementioned PROMs in a standardized battery which patients completed as a part of their treatment starting at clinical intake. As such, the collection of this COVID-19-specific and symptom-specific data spanning treatment provides the opportunity to assess temporal patterns in onboarding patient symptoms prior to/starting at intake, as well as the unique ability to evaluate interactions between impacts of the COVID-19 pandemic and patient psychiatric symptoms. The current study aims to analyze changes in the impact of COVID-19 on patient behavior and symptomatology, while further examining the relationship between CEC results and patient reports of anxiety, depression, and general psychological distress as seen in a midsized ambulatory psychiatric clinic. Researchers expected that COVID-19-related factors would negatively impact the mental health of the psychiatric population, both directly and via social determinants of health (SDOH) such as difficulties related to employment, schooling, housing, finances, taking care of children/family, attending doctor appointments, obtaining food, or other needed services/items. There was further expectation of rising psychological symptoms in the incoming patient population as they would not be expected to engage in or receive protective factors provided to ongoing psychiatric patients.

## Methods

### Participants and procedure

Participants in the current retrospective observational study comprise 842 new adult referrals at a hospital-based adult ambulatory psychiatric clinic in a midsized city in the southeastern United States who completed these measures as part of normative, yet virtually adapted, care during intake. While an author of this study is the recipient of a grant supporting their postdoctoral training, those funds are not specific to this research project and as such this investigator-initiated study did not have external funding support and was approved by the hospital system’s Institutional Review Board (IRB). The IRB deemed the current study IRB-exempt under the Department of Health and Human Services (DHHS) regulatory category 4(iii) as secondary research without consent (IRB # 20-905). Participants ranged in age from 18 to 90, the mean age being 43 years old (*SD* = 15.65). Participants were primarily female (71.02%; *n* = 598) (see Table 1). Participants were not required to provide ethnoracial belonging, so 28.15% (*n* = 237) did not report ethnicity. Of those that did report ethnic belonging, participants primarily identified as White or Caucasian (66.27%; *n* = 558), followed by Black or African American (4.04%; *n* = 34) and 1.54%; *n* = 13 “Other”.

**Table 1:** Patient demographic data

Race/ethnicity		*n*	%
	White or Caucasian	558	66.27%
	Black or African American	34	4.04%
	Other	13	1.54%
	Did Not Report	237	28.15%
	Total *(N)*	842	100%

Gender		*n*	%
	Female	598	71.02%
	Male	244	28.98%
	Total *(N)*	842	100%

Age		*n*	%
	18–22	30	3.56%
	23–27	63	7.48%
	28–32	71	8.43%
	33–37	67	7.96%
	38–42	60	7.13%
	43–47	54	6.41%
	48–52	48	5.70%
	53–57	52	6.18%
	58–62	62	7.36%
	63–67	45	5.34%
	68–72	32	3.80%
	73–77	17	2.02%
	78–82	6	0.71%
	83–87	1	0.12%
	88–92	2	0.24%
	Did Not Provide	232	27.55%
	Total *(N)*	842	100%

All participants completed one set of PROMs (CEC, PHQ-9, GAD-7, BASE-6) during intake. Subsequently, participants completed the CEC as an addition to the standard battery for 12 months, between April 2020 and March 2021. Individual responses of new adult patients at the clinic were scored for each measure, and patients’ aggregated scores were recorded and analyzed longitudinally by month. As the volume of patients able to complete an intake fluctuated widely during the first twelve months of the COVID-19 pandemic (See Supplemental Table 1), incoming patient scores were aggregated by month in order to capture trends in the overall mental well-being of patients following the declaration of a pandemic. This approach allowed researchers to review average incoming patient wellness scores in months such as July when less than a dozen patients completed intake, as well as December 2020 when over 150 patients completed intake. Aggregated monthly scores for the PHQ-9, GAD-7 and BASE-6 were determined by calculating the average scores on these measures for all new patients within that one-month period. Regarding the CEC data, patient scores were similarly averaged using each of the five index questions on the checklist. Details related to the CEC structure are outlined below.

### Measures

#### COVID-19 Event Checklist (CEC) [16]

The CEC is a self-report instrument that examines the occurrence of different events or situations that affect an individual’s physical/mental health and well-being as a consequence of the global pandemic. This measure was designed for adults who are at least 18-years-old and consists of five ‘yes or no’ index questions. Each of the five index questions has an accompanying checklist question that is only given if the initial answer is affirmative. The individual is then asked to indicate any items that are relevant to their experience. The CEC addresses five areas using an initial dichotomous outcome variable (yes/no): (1) exposure to or contact with someone with COVID-19 (“Have You (Or Someone You Know Well) Had Suspected Or Confirmed Exposure To Or Been Diagnosed With COVID-19?”), (2) use of preventive measures (“Have You Previously, Or Are You Currently, Engaged In Any Behaviors To Help Decrease Your Exposure And/Or Stop The Spread Of COVID-19?”), (3) Social Determinants of Health (SDOH) (“Over The Past Month, Have You Experienced Any Difficulties In Your Daily Life Or Work As A Result Of COVID-19?”), (4) negative emotional and mental health impact (“Over The Past Month, Have You Experienced A Worsening In Your Emotional State Or Psychological Well-Being As A Result Of COVID-19?”), and (5) use of positive coping skills (“Over The Past Month, Have You Engaged In Any Positive Coping Strategies To Deal With The COVID-19 Pandemic?”) [16]. To date, psychometric properties for this measure have yet to be published.

While researchers would usually prioritize the use of a psychometrically tested and validated measure, the CEC was created by a panel of recognized psychological experts in response to the newly declared pandemic and pragmatically employed within the first month of lockdown mandates. As a result of this rapid development in response to real-time events, the measure currently has little to no published psychometric data available. Per the creators of the CEC, initial organization of the five broad yes/no items was derived from a comprehensive literature review which was followed by professional consultation to affirm the validity of items as well as the pilot testing of measure items to ensure clarity:

An initial item pool was created following a comprehensive literature review (including a review of other recently published COVID-19-specific measures) and the generated items were sorted by domain. Refinement of the questions was then done in consultation with a team of psychologists and psychiatrists with a range of expertise and clinical experience. Finally, a small pilot study was conducted to further ensure the comprehensiveness and clarity of the CEC [16].

Via the nature of the scale and its exploration of individual experiences, measures of validity beyond corroboration from clinicians that aided in development of the measure would be nearly impossible to administer. As such, researchers elected to examine the measure’s reliability using patient data. Initial split-half reliability testing of inter-correlation using Cronbach’s Alpha was uninformative due to the nature of the CEC’s five primary yes/no questions which are not designed to be inter-related. Researchers then examined assessed average inter-item correlation (AIIC), which was more suitable for examining the dichotomous responses examined here. The AAIC of CEC responses collected within the ambulatory clinic’s incoming patients was 0.1219751, suggesting a positive, yet relatively modest level of average inter-item correlation.

#### Patient Health Questionnaire-9 (PHQ-9) [19]

The PHQ-9 is a 9-item self-report measure intended to evaluate and monitor the severity of depressive symptoms. The nine items align with the DSM’s diagnostic criteria of major depressive disorder (MDD) and have good internal consistency (α = 0.89) and reliability (*r* = 0.84). Evidence supports the PHQ-9 being reliable and valid in different settings with various populations, including psychiatric and general populations [4, 18].

#### Generalized Anxiety Disorder-7 (GAD-7) [30]

The GAD-7 was developed to capture the symptoms of generalized anxiety disorder in both adults and adolescent populations. The GAD-7 has demonstrated excellent internal consistency (α = 0.92) and test-retest reliability (*r* = 0.83). Previous studies demonstrates that the GAD-7 is a reliable and valid tool to screen for and evaluate anxiety symptoms that are discriminated with depressive symptoms [22, 25].

#### Brief Adjustment Scale-6 (BASE-6) [8]

The BASE-6 consists of six items and is a self-report questionnaire designed to measure adult clients’ general psychological adjustment and functioning. BASE-6 internal consistency ranges from good to excellent in nonclinical and clinical population samples (α = 0.87–0.93). Previous studies have demonstrated that the BASE-6 is psychometrically reliable, valid, and shows the same unidimensional construct and factorial invariance across different race/ethnic populations [17].

### Analysis plan

This retrospective, observational study examines CEC temporal patterns from initial outpatient psychiatric telemedicine appointments spanning April 2020–March 2021 (number of total months = 12) using aggregated patient data (See Supplemental Table 2). Regression slope analysis was conducted to assess the changes in affirmative responses to the five dichotomous CEC questions over time. Wilcoxon rank sum tests were then performed to assess non-aggregated PROMs measurements (i.e., PHQ-9, GAD-7, BASE-6) and primary dichotomous CEC survey questions (yes/no). As part of a larger published study [15], participants completing the CEC were initially compared to participants in a pre-COVID-19 period to examine the layered impacts of the COVID-19 pandemic on mental health as well as clinical services due to the necessitated transition to telemedicine.

## Results

### CEC temporal patterns

As participants of the current study were gathered during psychiatric intake at an adult outpatient ambulatory facility in an academic medical setting located in a midsize southeastern United States city, their numbers fluctuated widely during the first year of the COVID-19 pandemic, and variability in the number of incoming patient respondents completing PROMs each month (*N* = 12) was found. The real-world nature of this variability contributed to PROM completion ranging from regularly having over 100 patients submit data, to having less than 10 during a few months (July–September 2020). This variability of attendance and data is particularly notable in the decrease of affirmative responses to three of the CEC’s index questions during September 2020. While researchers are unable to provide definite reasons for this variability, these months coincide with noteworthy rates of COVID-19 transmission and hospitalizations as daily rates of new infections in the United States rose above 50,000 new cases per day in July 2020 [1, 33]. With the return to in-person public school in September 2020, the southeastern United States county in which this study was conducted saw new daily COVID-19 cases rise from approximately 5 per 100k residents up to 62 new cases of COVID-19 per 100k residents daily which may account for the low attendance reported during that period as observed in an ambulatory psychiatric clinic within a midsized city [2]. Inversely, the clinic saw intake numbers rise in December 2020 following the release of the first FDA-approved COVID-19 vaccines available to the public which may have increased patient ability or willingness to attend appointments.

**Fig. 1:**
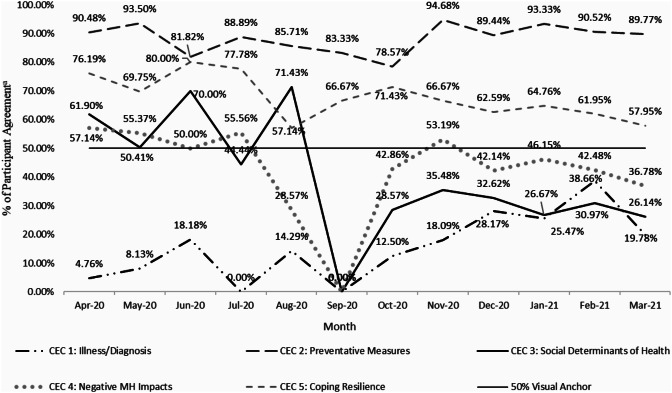
Patterns of intake CEC responses over the 12-months initiating the COVID-19 pandemic. ^a^Value indicated represents percentage of “yes” responses for each question per month by new clients during intake

During intake, patients were administered the standard battery of PROMs with the addition of the CEC, and on a monthly basis these intake scores were aggregated by measure and ultimately analyzed for temporal trends and patterns. As incoming patient PROM scores were collected, aggregated monthly CEC scores reported the percentage of participants responding in the affirmative to each of the five primary questions (See Supplemental Table 3). Researchers then conducted a regression analysis of the monthly CEC affirmative responses which provided slope values that were interpreted as changes in affirmative reports over time. The following results as seen in Fig. 1 represent a change in the percentage of patients responding “yes” to the inquiries. Incoming patient reports of suspected or confirmed COVID-19 exposure, as well as COVID-19 diagnosis increased significantly over the course of the study via the CEC (+ 2.29%, *p* = 0.001). Concurrently, incoming patients were less likely to report a negative impact of COVID-19 on SDOH from April 2020 to March 2021 (−3.53%, *p* = 0.034). Regarding the use of positive coping strategies to deal with the pandemic, participants reported significantly decreasing employment of these behaviors (*p* = 0.010) at a rate of −1.47% per month. Results suggest there was not a significant change in reported use of behaviors to mitigate exposure/spread of COVID-19 or in worsening emotional state or psychological well-being as a result of COVID-19 over the 12-month time period (see Fig. 1, Supplemental Fig. 1).

### Relationship between CEC and other PROMs

Prior to comparison with CEC results, PROMs data from PHQ-9, GAD-7 and BASE-6 were explored temporally, showing no significant differences in new patient symptom severity between the pre-COVID-19 period with similar enrollment (Nov 2019–Feb 2020) and COVID-19 pandemic (Mar 2020–Mar 2021) [15]. Simple statistics for these PROMs scores at intake are listed in Table 2. Comparisons of participants’ CEC scores with other PROMs demonstrated significant relationships between reported symptoms of depression, anxiety, and general psychological distress and some of the COVID-19-related questions (Table 3). Patients’ responses to the CEC questions investigating patients’ exposure to or contact with someone with COVID-19, and use of preventive measures, were not significantly correlated with their median scores on the BASE-6 (*p* = 0.78, *p* = 0.72), PHQ-9 (*p* = 0.60, *p* = 0.79), and GAD-7 (*p* = 0.80, *p* = 0.63).

**Table 2:** Descriptive statistics of PROMs at intake

Measure	*N* ^a^	Mean	Std dev	Sum	Minimum	Maximum
BASE-6	12	23.85	1.26	286.22	22.33	26.38
GAD-7	12	10.91	0.97	130.91	9.17	13.04
PHQ-9	12	11.26	1.09	135.10	9.35	13.45

^a^The total number of samples, *N*^a^, indicates the number of months in which new intake patients were assessed for the current sample

**Table 3:** PROMs results compared to CEC responses

CEC question	Response	BASE-6 [CI]	^*p*-value	GAD-7 [CI]	^*p*-value	PHQ-9 [CI]	^*p*-value
1a: exposure or diagnosis	Yes	22.0 [11.0, 32.0]	0.777	10.0 [4.0, 16.0]	0.792	11.0 [5.0, 18.0]	0.602
	No	23.0 [10.0, 32.0]		11.0 [4.0, 16.0]		10.0 [3.0, 17.0]	
2a: preventative measures	Yes	22.5 [11.0, 33.0]	0.178	10.0 [4.0, 16.0]	0.627	10.0 [3.0, 17.0]	0.790
	No	23.0 [10.0, 30.0]		11.0 [3.0, 16.0]		11.0 [3.0, 16.0]	
3a: SDOH	Yes	26.0 [15.0, 34.0]	< 0.000	12.0 [6.0, 17.0]	< 0.000	13.0 [6.0, 18.0]	< 0.000
	No	22.0 [9.0, 31.0]		9.0 [3.0, 15.0]		9.0 [2.0, 17.0]	
4a: mental wellness decrease	Yes	28.0 [16.0, 35.0]	< 0.000	12.0 [7.0, 17.0]	< 0.000	13.0 [7.0, 19.0]	< 0.000
	No	19.0 [7.0, 29.0]		7.0 [1.0, 14.0]		8.0 [1.0, 15.5]	
5a: positive coping	Yes	20.0 [9.0, 30.0]	< 0.000	8.0 [3.0, 15.0]	< 0.000	9.0 [3.0, 16.0]	< 0.000
	No	27.0 [17.0, 34.0]		12.5 [6.0, 17.0]		13.0 [6.0, 19.0]	

*Note P*-values were calculated from Wilcoxon rank-sum tests

BASE-6, GAD-7, and PHQ-9 are all represented as medians and interquartile ranges

^*p*-values represent statistical significance < 0.05

BASE-6 survey responses statistically differed depending on whether patients endorsed or denied a worsening in their emotional state or psychological well-being (*p* < 0.001), difficulties in patient daily life or work (SDOH) (*p* < 0.001), or use of positive coping strategies related to COVID-19 on the CEC (*p* < 0.001). Patients indicating a worsening in emotional state or psychological well-being related to COVID-19 reported a median BASE-6 score of 28.0 [IQR = 16.0, 35.0], while patients who did not reported a lower median BASE-6 score of 19.0 [IQR = 7.0, 29.0]. Regarding difficulties in daily life or work as a result of COVID-19, patients noting “yes” to this question had a higher median BASE-6 score (26.0 [IQR = 15.0, 34.0]) than those who responded “no” to this query (22.0 [IQR = 9.0, 31.0]). In addition, patients who reported using positive coping strategies to deal with the pandemic demonstrated a lower median BASE-6 score (20.0 [IQR = 9.0, 30.0]) than those who denied use of positive coping (27.0 [IQR = 17.0, 34.0]).

Similarly, patients’ median score on the GAD-7 significantly differed (*p* < 0.0001) depending on whether they endorsed difficulties related to SDOH (12.0 [6.0, 17.0]) or did not (9.0 [3.0, 15.0]). Acknowledgement of worsening emotional or psychological wellbeing resulting from COVID-19 was associated with significantly higher scores (*p* < 0.0001) on the GAD-7 (12.0 [7.0, 17.0]) compared to those who did not report experiencing this concern (7.0 [1.0, 14.0]). Patients who identified use of positive coping strategies reported a median GAD-7 of 8.0 [3.0, 15.0], and those who did not use positive coping had a higher median GAD-7 (12.5 [6.0, 17.0]). This difference in median GAD-7 scores was significant, *p* < 0.0001.

**Fig. 2:**
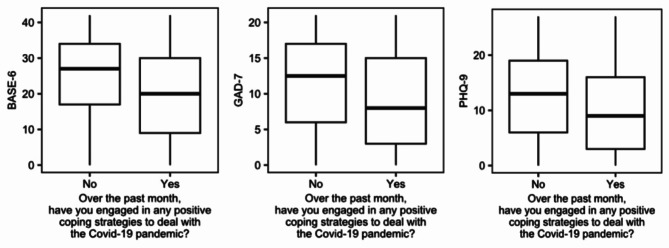
CEC question 5 (coping/resilience) and PROMs rank sum tests

Regarding patient’s responses to the PHQ-9, patients who reported “yes” to experiencing difficulties in daily life and work on the CEC had a higher median score (13.0 [IQR = 6.0, 18.0]) than those who responded “no” (9.0 [IQR = 2.0, 17.0]). PHQ-9 median responses also differed significantly for patients who endorsed a negative emotional and mental health impact of the pandemic (13.0 [IQR = 7.0, 19.0]) compared to those who did not (8.0 [IQR = 1.0, 15.5]). Patients identifying the use of positive coping skills on the CEC reported a lower median PHQ-9 score (9.0 [IQR = 3.0, 16.0]) than those who did not (13.0 [IQR = 6.0, 19.0]). Figure 2 contains plots demonstrating the differences in PROMs scores based on participants’ response to this query related to use of positive coping skills on the CEC (See Supplemental Figs. 2 and 3 for BASE-6 and PHQ-9 rank sum test box plots).

## Discussion

### CEC temporal patterns

The findings of this study indicate that patients reported more exposure to COVID-19 as the pandemic progressed. Along with this change, reports demonstrating an impact on work or daily life decreased over time. This outcome may be related to a shift of flows back into employment after higher unemployment rates that began in March 2020 [5]. The initial loss of income and benefits was followed by trends of stringent restrictions (i.e., isolation) generally easing, people returning to work with personal protective equipment, and fewer people losing jobs as the pandemic progressed. Additionally, governmental financial support was provided to individuals, families, and businesses in 2020, providing some relief [31].

Current results also indicate a decrease in the reported use of positive coping strategies over time. While understanding the exact mechanism of this change is beyond the scope of this study, this finding may be related to a decreased propensity to be intentional about coping as individuals became accustomed to the pandemic being a normal part of life. Research targeting stress, coping, and resilience during the early months of the pandemic indicated a decrease in early levels of distress and COVID-19-related distress over time [24]. The study findings also suggested that levels of overall psychological wellbeing remained similar to normal levels. Taken together, the authors concluded a resilient response to pandemic stress in their sample. Similar findings were reported in a study of the impacts of COVID-19 restrictions which found that both anxious and depressive symptoms had started to decline around the first 20 weeks of the pandemic [12]. These findings which suggest resilience and decreasing stress over time may explain the downward trend in reported positive coping found in the current study. Furthermore, the possible return to work for some patients may have also limited their ability to engage in coping strategies such as hobbies, family time, or being outdoors as frequently as they did during the first months of lockdown.

### Relationship between CEC and other PROMs

This study examined the relationship between CEC results and patient reports of anxiety, depression, and general psychological distress at intake. Patients in this study entering ambulatory psychiatric treatment that acknowledged a negative change in their SDOH reported higher scores on PROMs measuring anxiety, depression, and general psychological distress. These data are consistent with previous studies of the relationship between mental health symptoms and SDOH in adults with preexisting depression and anxiety. For instance, Alegría et al. [3] reported increased anxiety scores for patients with food insecurity and higher depression scores for patients with both food and utility insecurity.

Results indicate an association between scores on PROMs and the negative emotional impact of the COVID-19 pandemic. Meta-analytic data indicates that within the first 6 months of the COVID-19 pandemic, rates of depression, anxiety, posttraumatic symptoms similar to those assessed in the current study increased for up to 77% of individuals experiencing mental illness and up to 94% of individuals without mental illness [10]. When examining reports of worsening emotional and mental health resulting from COVID-19 via the CEC, the current data shows that incoming patients reporting such experiences displayed significantly higher psychological distress, depression, and anxiety symptoms on the respective administered PROMs than those who did not. Since the CEC was designed shortly before the collection of the present data, the current findings support the validity of the novel measure and its ability to evaluate the presence of clinically meaningful negative mental health impacts indicative of elevated depressive, anxious, and psychological distress symptoms. Results also suggest an association between PROM scores and worsening mental health symptoms.

During the evaluation of incoming patients’ employment of positive coping skills in response to the COVID-19 pandemic, participants who reported using such positive coping skills reported fewer symptoms of depression, anxiety, and psychological distress than those who failed to engage in coping. The displayed relationship affirms our findings and yet again emphasizes the protective effects of positive coping skills on psychological wellbeing in the form of depressive and anxious symptomatology, as well as reported psychological distress. Though researchers are unable to confirm or validate such an assumption in the current sample, per the specialty nature of any psychiatric clinic, most new and incoming clients are likely referred to psychiatric services through some sort of previous or ongoing mental health treatment and are likely to have access or exposure to these protective factors [7, 23, 32].

### Limitations

This study has several limitations to note. First, this sample was comprised of patients entering outpatient psychiatric care in the southeastern United States, allowing for limited generalizability. Data was gathered via cross-sectional design and was observational in nature; thus, the study did not track changes in individual patients over time and allows for limited interpretations due to the correlational nature of the methodology. There was variability in the number of patients completing the CEC from month to month, thus impacting the probability of error. Furthermore, while this research employed commonly used assessment tools to measure psychological symptomatology (GAD-7, PHQ-9, and BASE-6), the CEC is a new inventory. The current study is unique in that, to our knowledge, research using the CEC has not been published. Indeed, this novelty is counterbalanced by the lack of literature demonstrating its use and utility. The CEC was developed for utilization in response to the COVID-19 crisis employing a comprehensive literature review, expert consultation, and piloting. While the need for pragmatism on the part of the checklist creators as a function of the rapidly emerging and developing nature of the pandemic is appreciated, the absence of established validity and psychometric properties is a limitation of this study. The CEC allows for significant COVID-19-related information to be collected, but general crisis events checklists may be more optimal choices in future studies as they allow for comparison between crisis events.

### Implications

Future evaluation of CEC data can add qualitative richness and item-level rate of reporting information with respect to patients’ pandemic-related experience. Future analyses may be able to distinguish the specific actions or behaviors associated with temporal trends noted in this study, including the exploration of mediating or moderating effects. Lastly, the nature of data collection and analyses utilized does not allow for the opportunity to definitively discuss causal inferences in this research. Literature examining behaviors, outcomes, and mental health during significant crisis events like the COVID-19 pandemic will benefit greatly from employing mechanisms to allow for investigating causal mechanisms of change.

## Conclusion

The current study provides insight into the association between the COVID-19 event, pandemic-related behaviors and outcomes, coping, and mental health symptoms. The CEC alone allows for a multifaceted investigation of the impacts of COVID-19 on patients, and strategies they used to manage during the pandemic. Using additional PROMs, this work captured patients’ self-reported depression, anxiety, and general psychological functioning beyond pandemic-related distress alone, as well as the relationship between COVID-19-related factors and mental health. The routine use of PROMs facilitated the ability to view population trends over several months during the pandemic, thus capturing how patients entering psychiatric care responded to the pandemic.

Overall, these results provide evidence of the relationship between SDOH, coping skills, mental health symptoms, and the impacts of the COVID-19 pandemic. The study highlights the importance of promoting the use of positive coping skills to mitigate the negative mental health effects of pre-existing conditions such as anxiety and depression, during broad environmental stressors.

## Electronic supplementary material

Below is the link to the electronic supplementary material.

Supplementary Material 1

## Data Availability

The datasets generated and analyzed during the current study are not publicly available because they contain information gathered during confidential medical visits. Data absent of personally identifiable information are available from the corresponding author on reasonable request.

## Abbreviations

MBC: Measurement Based Care
PROM: Patient-Reported Outcome Measures
CEC: COVID-19 Events Checklist
GAD-7: General Anxiety Disorder-7
PHQ-9: Patient Health Questionnaire-9
BASE-6: Brief Adjustment Scale-6
SDOH: Social Determinants of Health
