# Supplementary material for: Assessing the impact of COVID-19 on outpatient psychiatric population well-being and symptomology utilizing COVID-19 Events Checklist (CEC) and Measurement Based Care
Source: J Patient Rep Outcomes. 2024 Nov 21;8:135. doi: 10.1186/s41687-024-00802-z (PMC11582237; doi:10.1186/s41687-024-00802-z)

**CEC Supplemental Table & Figures**

**Supplemental Table 1**

*Monthly Patient Intake Totals*

| **Month** | **Clients *(N)*** |
| --- | --- |
| Apr-20 | 22 |
| May-20 | 123 |
| Jun-20 | 12 |
| Jul-20 | 9 |
| Aug-20 | 7 |
| Sep-20 | 7 |
| Oct-20 | 18 |
| Nov-20 | 104 |
| Dec-20 | 172 |
| Jan-21 | 129 |
| Feb-21 | 136 |
| Mar-21 | 103 |
| Total | 842 |

**Supplemental Table 2**

*Pairwise Sample Sizes for Testing*

|  | BASE-6 | GAD-7 | PHQ-9 |
| --- | --- | --- | --- |
| 1A | 714 | 718 | 708 |
| 2A | 708 | 712 | 702 |
| 3A | 703 | 707 | 697 |
| 4A | 700 | 703 | 694 |
| 5A | 698 | 701 | 693 |

**Supplemental Table 3**

*CEC Response Affirmative Response Monthly Data*

| Month | CEC 1: Illness/Diagnosis | CEC 2: Preventative Measures | CEC 3: Social Determinants of Health | CEC 4: Negative MH Impacts | CEC 5: Coping/Resilience |
| --- | --- | --- | --- | --- | --- |
| Apr-20 | 5% | 90% | 62% | 57% | 76% |
| May-20 | 8% | 93% | 50% | 55% | 70% |
| Jun-20 | 18% | 82% | 70% | 50% | 80% |
| Jul-20 | 0% | 89% | 44% | 56% | 78% |
| Aug-20 | 14% | 86% | 71% | 29% | 57% |
| Sep-20 | 0% | 83% | 0% | 0% | 67% |
| Oct-20 | 13% | 79% | 29% | 43% | 71% |
| Nov-20 | 18% | 95% | 35% | 53% | 67% |
| Dec-20 | 28% | 89% | 33% | 42% | 63% |
| Jan-21 | 25% | 93% | 27% | 46% | 65% |
| Feb-21 | 39% | 91% | 31% | 42% | 62% |
| Mar-21 | 20% | 90% | 26% | 37% | 58% |

**Supplemental Figure 1**

**Fig. 1** *CEC Trendlines over 12 months*


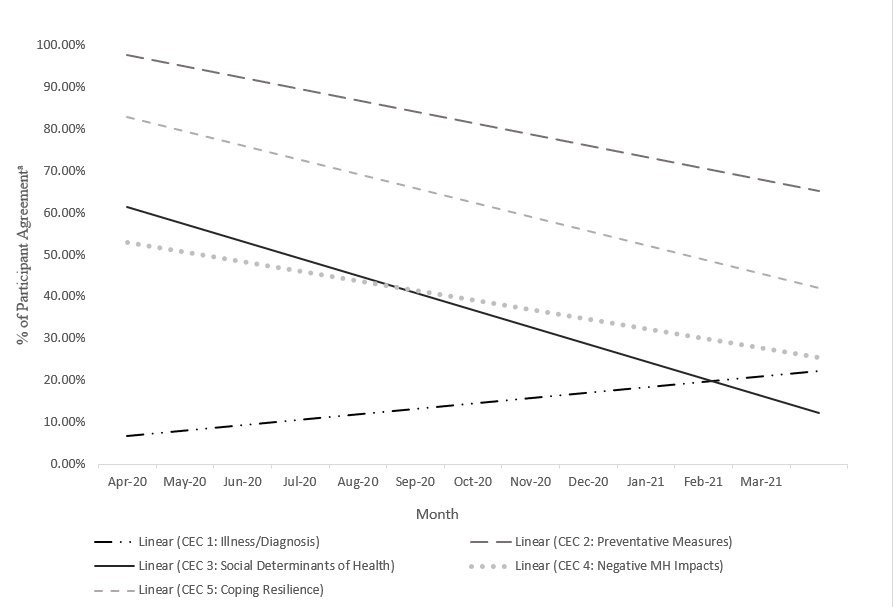


**Supplemental Figure 2**

**Fig 2.** *CEC and BASE-6 Rank Sum Tests*


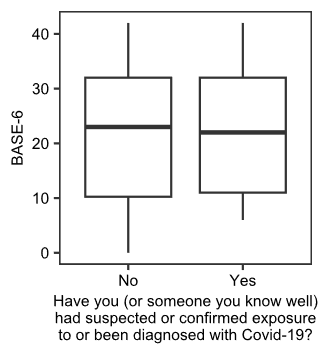

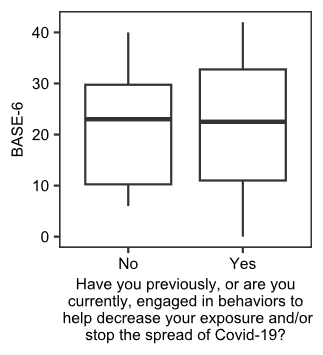

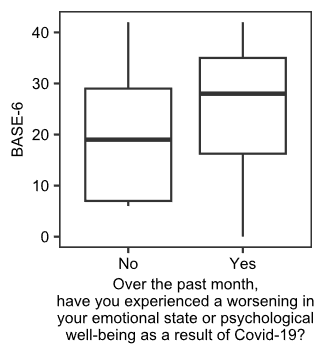

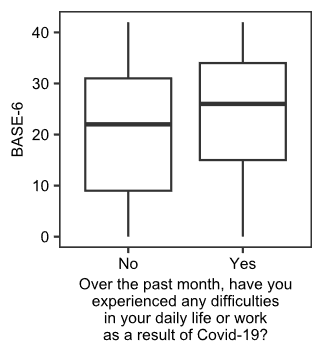


**Supplemental Figure 3**

**Fig 3.** *CEC and PHQ-9 Rank Sum Tests*


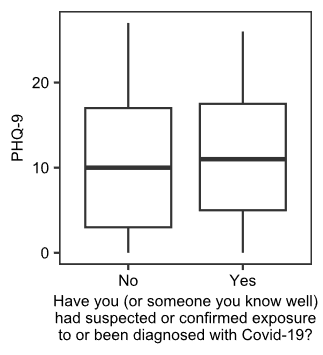

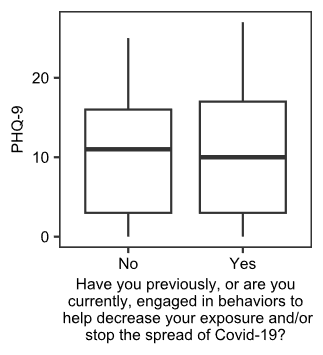

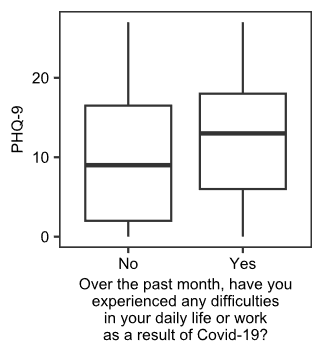

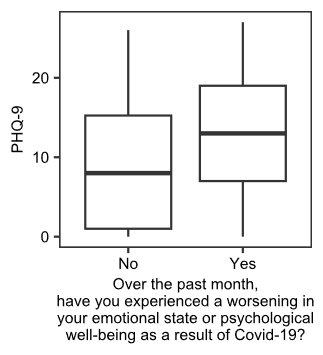

Supplement: Supplementary file 1 — Supplementary Material 1 [file 41687_2024_802_MOESM1_ESM.docx]
